# Differential Glycemic Effects of Low- versus High-Glycemic Index Mediterranean-Style Eating Patterns in Adults at Risk for Type 2 Diabetes: The MEDGI-Carb Randomized Controlled Trial

**DOI:** 10.3390/nu14030706

**Published:** 2022-02-08

**Authors:** Robert E. Bergia, Rosalba Giacco, Therese Hjorth, Izabela Biskup, Wenbin Zhu, Giuseppina Costabile, Marilena Vitale, Wayne W. Campbell, Rikard Landberg, Gabriele Riccardi

**Affiliations:** 1 Department of Nutrition Science, Purdue University, 700 West State St., West Lafayette, IN 47907, USA; robbergia@gmail.com; 2 Diabetes, Nutrition and Metabolism Unit, Department of Clinical Medicine and Surgery, Federico II University, 80138 Naples, Italy; rgiacco@isa.cnr.it (R.G.); giuseppina.costabile@unina.it (G.C.); marilena.vitale@unina.it (M.V.); gabriele.riccardi@unina.it (G.R.); 3 Institute of Food Science, National Research Council, 83100 Avellino, Italy; 4 Department of Biology and Biological Engineering, Food Science and Nutrition, Chalmers University of Technology, 41296 Gothenburg, Sweden; therese.hjorth@chalmers.se (T.H.); izabela.biskup@chalmers.se (I.B.); rikard.landberg@chalmers.se (R.L.); 5 Department of Statistics, Purdue University, West Lafayette, IN 47907, USA; zhu633@purdue.edu

**Keywords:** Mediterranean diet, metabolic syndrome, metabolic health, impaired glycemic control, metabolic risk factors, insulinemia, glycemic variability, continuous glucose monitoring, oral glucose tolerance test, meal glucose tolerance test

## Abstract

A Mediterranean-style healthy eating pattern (MED-HEP) supports metabolic health, but the utility of including low-glycemic index (GI) foods to minimize postprandial glucose excursions remain unclear. Therefore, we investigated the relative contribution of GI towards improvements in postprandial glycemia and glycemic variability after adopting a MED-HEP. We conducted a randomized, controlled dietary intervention, comparing high- versus low-GI diets in a multi-national (Italy, Sweden, and the United States) sample of adults at risk for type 2 diabetes. For 12 weeks, participants consumed either a low-GI or high-GI MED-HEP. We assessed postprandial plasma glucose and insulin responses to high- or low-GI meals, and daily glycemic variability via continuous glucose monitoring at baseline and post-intervention. One hundred sixty adults (86 females, 74 males; aged 55 ± 11 y, BMI 31 ± 3 kg/m^2^, mean ± SD) with ≥two metabolic syndrome traits completed the intervention. Postprandial insulin concentrations were greater after the high-GI versus the low-GI test meals at baseline (*p* = 0.004), but not post-intervention (*p* = 0.17). Postprandial glucose after the high-GI test meal increased post-intervention, being significantly higher than that after the low-GI test meal (35%, *p* < 0.001). Average daily glucose concentrations decreased in both groups post-intervention. Indices of 24-h glycemic variability were reduced in the low-GI group as compared to baseline and the high-GI intervention group. These findings suggest that low-GI foods may be an important feature within a MED-HEP.

## 1. Introduction

Type 2 diabetes is a dire metabolic condition that has a profound impact on the estimated ~400 million individuals afflicted worldwide [1]. Without a rapid and robust response, type 2 diabetes is projected to continue along this course of precipitous increases in cases during the upcoming decades [1,2]. The deleterious effects of type 2 diabetes are further magnified when its contributions towards cardiovascular disease, the leading cause of mortality in Western nations, are considered [3,4]. Given the societal burden, research investigating the potential for lifestyle and dietary interventions for those who are at risk of developing type 2 diabetes, must be fully leveraged if there is to be hope in slowing the steep rise in cases [5].

Postprandial glycemia may contribute as much or more than fasting blood glucose to the pathogenesis of impaired insulin sensitivity and insulin secretion seen in the progression towards type 2 diabetes [6,7]. Indeed, the loss of postprandial glucose control often manifests before derangements in fasting indices, along the path towards type 2 diabetes in at-risk individuals [8]. Therefore, strategies to attenuate postprandial glucose excursions may be of particular importance in the effort to reduce the global burden of disease. Proposed 40 years ago, the glycemic index (GI) of carbohydrate-containing foods is posited to play a substantial role in postprandial glucose excursions [9]. However, there is still not a consensus on the relevance and utility of GI in non-diabetic people [10], particularly in the context of a healthy eating pattern (HEP) where other health-promoting dietary factors may take precedence [11]. It is unclear whether differences in postprandial glucose and insulin responses that are induced acutely by various carbohydrate foods may disappear after a few weeks due to compensatory mechanisms operative in non-diabetic people [12].

Consuming a Mediterranean-style (MED) HEP is supported by considerable evidence suggestive of a reduced risk of type 2 diabetes development [13,14]. The preponderance of the evidence is observational in nature, with relatively little experimental evidence, particularly outside of the geographical Mediterranean region, supporting this HEP. Fat quality, dietary fiber, and different bioactive compounds have been emphasized as the core elements behind the observed health effects of a MED-HEP [15]. However, to the best of our knowledge, there have been no studies assessing the relative contributions of high- versus low-GI foods to improvements in indices of glucose control and cardiometabolic health in the context of a MED-HEP.

Therefore, we conducted a 12-week, multi-center, randomized, controlled intervention assessing the potential differential effects of iso-caloric, weight-maintaining, high- versus low-GI MED-HEPs on indices of glucose control among participants at risk for developing type 2 diabetes. We hypothesized that the low-GI group would present with lower postprandial glucose and insulin responses relative to the high-GI group at baseline and that these differential responses would be maintained at the end of the dietary intervention. Further, we hypothesized that the low-GI group would present with improvement in indices of glycemic variability from baseline to post-intervention, relative to the high-GI group.

## 2. Materials and Methods

The MEDGI-Carb trial is an international multi-center randomized, controlled, parallel-group, 15-week trial including a 3-week baseline period followed by 12 weeks of controlled dietary intervention. This research study was initiated in January 2018 and the trial continued through December 2019. This study was conducted at three centers: (1) Federico II University, Naples, Italy (2) Chalmers University of Technology, Gothenburg, Sweden, and (3) Purdue University, West Lafayette, IN, USA. The study protocol was approved by the institutional review boards at Purdue University and Federico II University and by the Swedish Ethical Review Authority. This study was registered in the public trial registry Clinicaltrials.gov (accessed on 7 December 2021) as NCT03410719. Detailed descriptions of study protocols for all centers are published [16].

### 2.1. Experimental Design

During the 12-week intervention period, subjects consumed a controlled, iso-energetic, weight-maintenance diet and were instructed to consume intervention-specific foods to achieve a low-glycemic or high-glycemic MED-HEP (low-GI or high-GI, respectively). Outcome measurements were obtained on standardized testing days to determine markers of glucose homeostasis by completion of an eight-hour meal glucose tolerance test (MGTT), including both breakfast and lunch resembling food choices of the assigned diet, an oral glucose tolerance test (OGTT), and 24-h continuous glucose monitoring on separate days at baseline and post-testing. Primary outcomes are postprandial plasma glucose and insulin responses during the MGTT; secondary outcomes include fasting and OGTT plasma glucose and insulin; blood HbA_1c_; and indices of 24-h glycemic variability.

### 2.2. Eligibility Criteria

The eligibility criteria were designed to select middle-aged and older adults at risk for developing type 2 diabetes. Therefore, adults with a waist circumference >102 cm (males) or >88 cm (females) and one additional trait of the metabolic syndrome, according to the National Cholesterol Education Program’s Adult Treatment Panel III [17], were recruited. The additional traits could include blood pressure >130/85 mmHg or taking medication to control high blood pressure; fasting plasma glucose 5.6–7.0 mmol/L; fasting triglycerides 1.7–4.5 mmol/L; HDL < 1.0 mmol/L (males) or <1.3 mmol/L (females). A member of the research team at each of the three testing sites, who was not involved in data collection or analysis, generated the random allocation sequence and assigned subjects to the interventions. Each subject was randomly assigned to one of two dietary groups using either a stratified block pattern (Italy & US; *n* = 8, 10 blocks; 4 randomized to each group per block of 8, using an online randomization plan generator (http://randomization.com/ (accessed on 31 January 2022) or a mixed size of the block pattern (Sweden; 4, 6, and 8 subjects per block in random block order, using Rstudio software version 2.4.0 (RStudio, Boston MA, USA) with package ‘blockrand’ version 1.3)). The randomization code remained unrevealed until all participant testing and analyses of samples for a priori primary outcomes were completed. Full inclusion criteria details, recruitment, and consent procedures can be found elsewhere [16].

### 2.3. Dietary Control

During the 3-week baseline period, all subjects consumed their habitual, self-chosen, unrestricted diets. Throughout the 12-week intervention period, each subject was counseled to follow their assigned iso-caloric MED-HEP using a combination of prescribed menus (breakfast, lunch, and snacks) and an item-specific version of the ‘Dinner Recipe Builder’ for dinner. The Dinner Recipe Builder is a mechanism to self-efficacy by which participants were given the flexibility to mix and match ingredients, while still following a MED-HEP. The two group-specific diet plans contained primarily the same foods and beverages in their MED-HEPs, except for substitutions of major sources of starch in the meals. All participants were advised to consume the same quantity of metabolizable carbohydrate (270 g/d) and fiber (35 g/d). Higher or lower energy content was achieved through the modulation of dietary fat and protein. One-half of daily carbohydrate (135 g) was the same for both the low- and high-GI groups, including the carbohydrates in fruits, vegetables, and other foods that all subjects consumed. The other one-half of the daily carbohydrate intake (135 g) was specific to the low-GI and high-GI groups. Specifically, 135 g of carbohydrate in the low-GI group came from foods with GI values < 55, while 135 g of carbohydrate in the high-GI group came from foods with GI values > 70. These GI cut-points correspond with those indicative of low-GI foods (<55) and high-GI foods (>70) [18]. The intervention-specific carbohydrates were distributed as 35 g at breakfast, 40 g at lunch, and 60 g at dinner. Complete descriptions of dietary controls can be found elsewhere [16]. Briefly, all participants were provided with group-specific instructions on the quantities of specific foods to consume. They were also given selected food items to use for their meals (high-GI jasmine rice, potato, mashed potatoes, couscous, wholegrain bread, and rusks; low-GI pasta, brown rice, flatbread, all bran, and wheat, plus rye bread and seeds). Dietary counseling was given bi-weekly and included group dinner meal preparation sessions.

### 2.4. Postprandial Assessments

Visits for clinical assessments at baseline and post-intervention included an 8-h MGTT and a 2-h OGTT (Supplemental Figure S1). Prior to all testing days, participants were instructed not to eat or drink anything (except a small amount of water) from 22:00 h the evening before the visit. Participants were counseled to refrain from vigorous physical activity for 48 h prior to testing days, avoid alcohol 24 h prior to testing days, and avoid caffeinated beverages the morning of testing days. After arriving at the testing facilities, participants would be seated in a chair/bed to rest. A catheter was placed in an antecubital vein and remained in place for the remainder of the testing day. Blood pressures were taken in duplicate after 15 min of rest.

For the OGTT and MGTT, double baseline fasting blood samples were collected at the minus 15-min time-point and the minus 5-min time-point following 15 min of rest (pooled ((mean value)) and denoted as time-point −10). The 75 g glucose drink (OGTT) or test meal (MGTT) were consumed at time-point 0. Full test meal contents are provided in Supplemental Table S1. Each participant consumed a test meal consistent with the composition of the assigned diet, at baseline, and at the end of the intervention. During the OGTT, blood samples were collected at 60 min and 120 min after consumption of the test glucose beverage. Subjects were not permitted additional fluid consumption during the test. During the MGTT, blood samples were collected immediately following the test breakfast meal (time-point +15) and then at intervals progressing from 15 min to one hour from time-points +30, 45, 60, 90, 120, 180, and 240. The second test meal was provided following the time-point +240 blood draw. The blood draw pattern of timing was repeated after the second meal was consumed.

### 2.5. Continuous Glucose Monitoring

Medtronic iPro2 Professional continuous glucose monitoring devices (Northridge, CA, USA) were used to obtain 24-h interstitial glucose concentrations in 5-min intervals during the baseline and post-testing weeks. Data were entered into the EasyGV platform (University of Oxford, Oxford, England) for calculation of the relevant indices of glycemic variability in non-diabetic individuals (mean amplitude of glucose excursions; continuous overall net glycemic action; mean absolute glucose; and lability index).

### 2.6. Blood Collection and Analysis

During the 12-week intervention period, subjects consumed a controlled, iso-energetic, weight-maintenance diet and were instructed to consume intervention-specific foods to achieve a low-glycemic or high-glycemic MED-HEP (low-GI or high-GI, respectively). Complete descriptions of blood sample collection analyses can be found elsewhere [16]. Briefly, blood samples were obtained from an antecubital vein and placed in tubes containing a clot activator to obtain serum or sodium/lithium heparin to obtain plasma. Serum tubes were held at room temperature for at least 15 min and then centrifuged at 4000× *g* at 4 °C for 15 min (3000× *g* at 4 °C for 10 min in Sweden). EDTA plasma, serum, and heparinized plasma samples were immediately refrigerated/kept on ice, processed, and aliquoted into microtubes. Plasma and serum aliquots were frozen at −20 °C within 2 h of sample collection, stored at this temperature for a maximum of one week, and then stored at −80 °C until thawed for analysis. EDTA plasma samples were used to assess insulin and glucose concentrations. All samples were analyzed at the end of the study to minimize batch effects.

### 2.7. Statistical Analyses

The primary analyses followed the intention-to-treat plan. A repeated measures linear mixed model was used to model the main effects of group, time, and group × time interactions as well as the effect of the study center. Least square means for outcomes of interest were calculated via the *LSMESTIMATE* statement using the *PROC MIXED* procedure in SAS statistical software version 9.4 (SAS Institute, Cary, NC, USA). Power calculations indicated that 180 subjects total (90 low-GI, 90 high-GI; 60 from each testing center) would provide greater than 80% power to detect a 30% differential response between the dietary interventions for the primary endpoint (postprandial insulin) with similar variation reported in the study by Giacco et al. [19]. Postprandial glucose and insulin averages were assessed by setting the minimum-reported value of the participant as the baseline, whereby all subsequent values were firstly subtracted from the minimum values, and then were averaged. The same standard was used in the calculation of postprandial glucose and insulin areas under the curve (AUC, using the trapezoidal rule). This method minimizes the conceptual errors associated with large glucose excursions washing out and not being represented in postprandial assessments, i.e., high glycemic variability where large peaks (hyperglycemia) are followed by large depressions below fasting concentrations (hypoglycemia). Calculation with the AUC_min_ method consistently resulted in the lowest intra- and inter-individual coefficients of variation in previous assessments of postprandial glycemia [20].

When confronted with missing data from postprandial assessments, multiple imputation using chained equation procedures were followed in accordance with Rubin’s rules [21] to combine the statistical results from each individual imputed complete data. Covariates accounted for in all statistical models included BMI, waist circumference, age, sex, smoking status (smoker/non-smoker), and testing center (Italy, Sweden, or USA). When no significant group × time interaction was observed, data from both groups were pooled to assess the overall effect of the dietary intervention. Significance was set at *p* < 0.05.

## 3. Results

**Figure 1:**
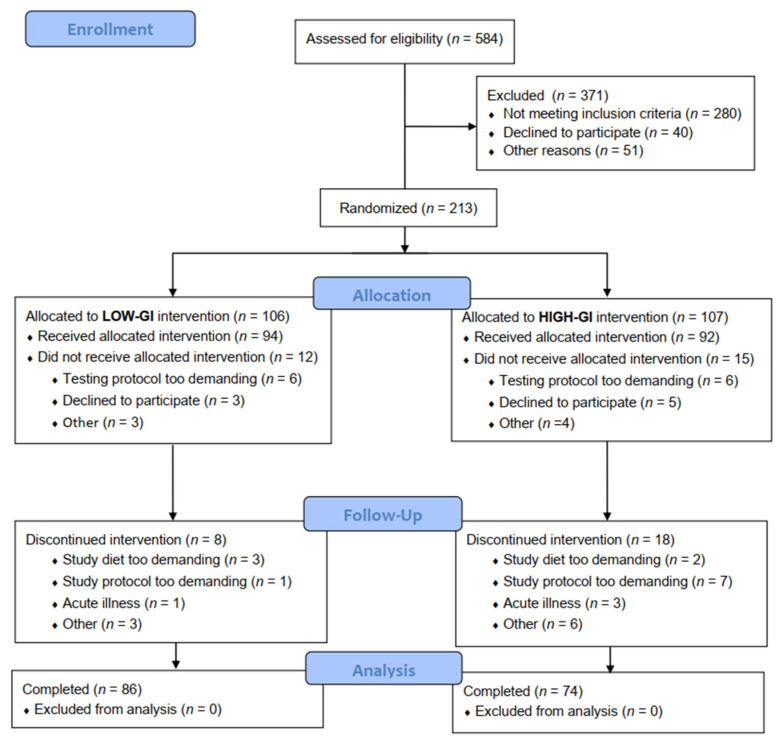
CONSORT participant flow diagram.

The CONSORT participant flow diagram is presented in Figure 1. During the clinical testing phase (February 2018 to December 2019), 584 participants were screened for eligibility. Of the initially enrolled 213 participants, 27 (low-GI; *n* = 12, high-GI; *n* = 15) dropped out prior to commencing the dietary intervention, and 26 (low-GI; *n* = 8, high-GI; *n* = 18) dropped out during the dietary intervention, resulting in 160 (low-GI; *n* = 86, high-GI; *n* = 74) participants completing the dietary intervention.

### 3.1. Baseline Characteristics

There were 119 female participants and 94 male participants (Table 1). In our sample of adults at risk for type 2 diabetes, all participants presented with elevated waist circumference (by design). Forty-four percent of participants had two traits of the metabolic syndrome, 37% had three traits (and thus are classified as having the metabolic syndrome), 16% presented with four traits, and 3% had all five traits. Elevated blood pressure was the most common secondary trait (60%), followed by elevated fasting glucose (46%), low HDL (38%), and elevated triglycerides (33%).

### 3.2. Dietary Composition

At baseline, the two groups did not present with differences in any dietary features (Supplemental Table S2). Post-intervention, both groups reduced their intakes of total saturated and polyunsaturated fat, while their intakes of fiber, monounsaturated fat, and carbohydrates increased. Post-intervention there was no difference in energy or nutrient composition between the low- and high-GI groups. Targeted differences in the glycemic index were achieved with average glycemic index values of 46.8 ± 3.1 vs. 66.2 ± 4.7 reported in the low- and high-GI groups, respectively.

### 3.3. Postprandial MGTT Glucose and Insulin Responses

**Figure 2:**
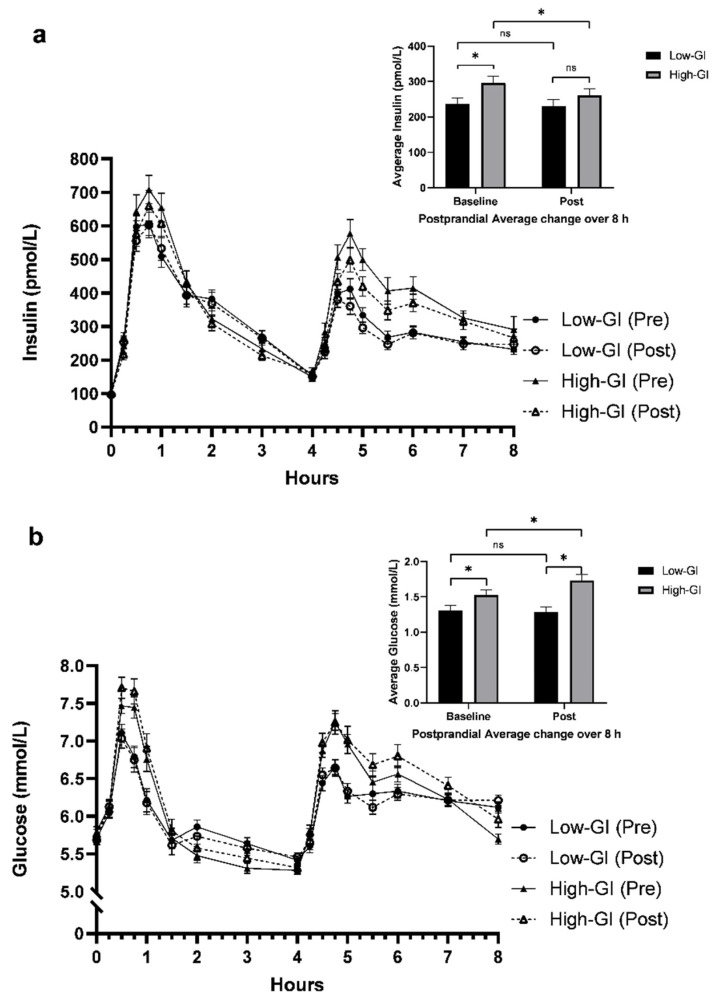
Insulin (**a**) and glucose (**b**) responses to a low-GI and high-GI 8-h meal glucose tolerance test at baseline and after a 12-week dietary intervention. Inset bar graphs display average postprandial insulin and glucose elevations above fasting concentrations over the 8-h period. Data are means ± SEM. * Statistically significant, *p* < 0.05. ns, no significance.

Postprandial insulin and glucose responses to the 8-h MGTT are presented in Figure 2. Average postprandial insulin was greater after the high-GI test meals compared to the Low-GI test meals at baseline (*p* = 0.004), but this difference was no longer present post-intervention (*p* = 0.17). From baseline to post-intervention, postprandial insulin decreased in the high-GI group, but not in the low-GI group (group × time; Δ −30.6 ± 15.3 pmol/L; *p* = 0.046). Postprandial glucose was greater (~17%) after the high-GI vs. the Low-GI test meals at baseline (*p* = 0.02), and more so post-intervention (35%; *p* < 0.001). This greater difference between low- and high-GI groups post-intervention was attributable to increases in average postprandial glucose over the 12 weeks in the high-GI group (Δ 0.2 ± 0.1 mmol/L; *p* = 0.03). Similar to postprandial insulin responses, glucose responses in the low-GI group were unchanged after the intervention. Overall, there were more robust differential effects of GI between the groups at the lunch meals, while glucose and insulin responses were less different after the breakfast meals (Supplemental Figure S2). Postprandial glucose and insulin AUC results were comparable to those seen for postprandial averages (Supplemental Figure S3).

Our study was not designed to determine differences in glycemic responses based on sex or race/ethnicity per se. Most participants were Caucasian non-Hispanic (*n* = 158, 99%), therefore, no meaningful post hoc analyses can be conducted. Sex effects were detected during modeling postprandial insulin, but not postprandial glucose responses.

### 3.4. 24-h Glycemic Variability

**Figure 3:**
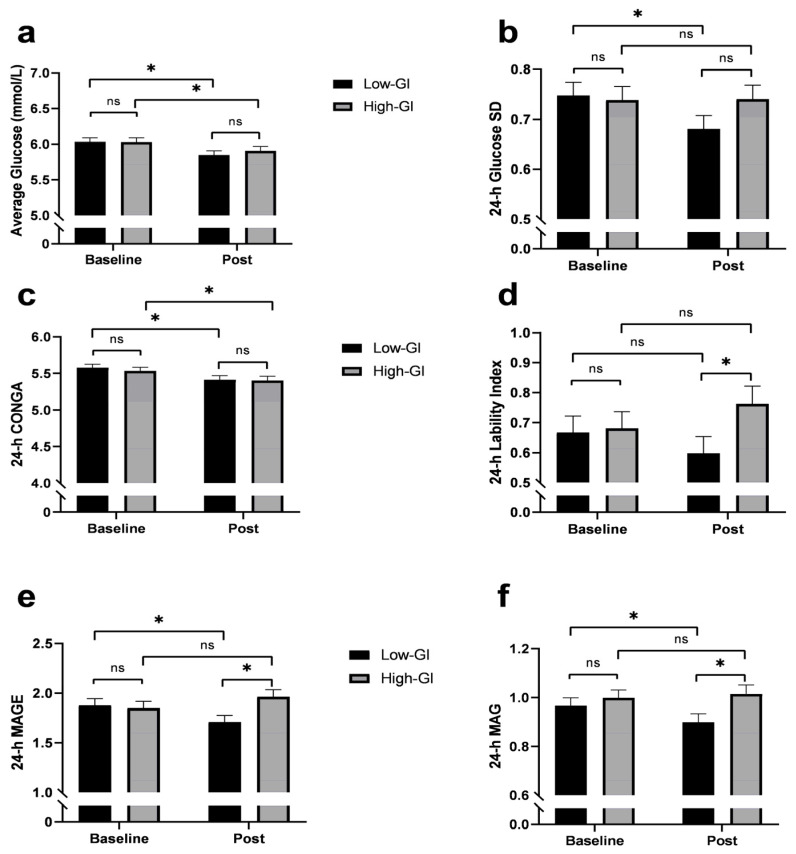
24-h continuous glucose monitor-derived measures of glycemic variability at baseline and after a 12-week dietary intervention. (**a**) Average 24-h glucose concentration, (**b**) standard deviation (SD), (**c**) continuous overall net glycemic action (CONGA), (**d**) lability index, (**e**) mean amplitude of glucose excursions (MAGE), and (**f**) mean absolute glucose (MAG). Presented data are means ± SEM. * Statistically significant, *p* < 0.05. ns, no significance.

At baseline, all 24-h continuous glucose monitoring variables were not different between the groups (Figure 3). Average 24-h glucose concentrations decreased in both groups, with indices of glycemic variability improving in the low-GI group, but not in the high-GI group. The low-GI group presented with reductions in 24-h standard deviation, mean amplitude of glucose excursions, and mean absolute glucose. Lability index (*p* = 0.04), mean amplitude of glucose excursions (*p* < 0.01), and mean absolute glucose (*p* = 0.02) were lower in the low-GI group compared to the high-GI group post-intervention.

### 3.5. Fasting and OGTT Glucose and Insulin Responses

There were no differences in 1-h or 2-h glucose (Supplemental Figure S4a) and insulin (Supplemental Figure S4b) between the low- and high-GI groups at either baseline or post-intervention. Irrespective of GI, glucose responses, but not insulin, decreased over the course of the intervention (pooled analysis; *p* = 0.02). The GI of the MED-HEP did not influence changes over time in the fasting risk factors for type 2 diabetes, including fasting glucose, insulin, HOMA-IR, and HbA_1c_ (Supplemental Table S3).

## 4. Discussion

This study demonstrated that consuming a Mediterranean-style eating pattern with low-GI foods reduces daily glycemia and indices of glycemic variability in adults at risk for type 2 diabetes. As hypothesized, postprandial glycemia was lower after the low-GI test meals relative to the high-GI test meals at baseline, and the magnitude of this difference increased during the dietary intervention. This widened difference was primarily attributable to increases in the high-GI group’s postprandial glucose, while the low-GI group’s glucose responses were not different between baseline and post-intervention. This suggests that a diet based on high-GI foods, including 50% of carbohydrates from foods with GI values > 70, significantly increases the postprandial glucose response, not only in an acute setting but also in the long term despite adherence to an overall healthy eating pattern.

Contrary to our hypothesis, while postprandial insulin was lower in the low-GI group compared to the high-GI group at baseline, this difference was no longer present post-intervention; in fact, postprandial insulin decreased at post-intervention more in the high-GI group relative to the low-GI group. However, this decline in the postprandial insulin occurred only at breakfast, while plasma insulin concentrations after lunch remained significantly higher during the intervention in the high-GI group. Indeed, the difference in postprandial insulin at lunch was more than 50% higher in the high-GI group. Postprandial plasma insulin concentrations are generally considered a marker of increased cardiovascular risk and therefore the increased postprandial insulin concentrations after lunch might be considered as a potential untoward effect of the high GI diet [22].

In contrast to the clear benefits of a low-GI diet over a high-GI diet regarding its effects on postprandial glucose metabolism, we detected no improvement in fasting glucose, OGTT, or HbA_1c_. This was unsurprising, as fasting plasma glucose concentrations are typically unaffected by weight-maintaining dietary interventions, particularly when they are not clearly elevated [23]. In addition, HbA1c is an imprecise marker of glucose control for values below 5.5% (37 mmol/mol), which is the range in which most of our participants were included [24]. Regarding OGTT responses, improvements in this marker are only reported in long-term weight-loss interventions in individuals with diagnosed impaired glucose tolerance [25].

Our study results are concordant with the outcomes of one previous study [26] where individuals with prediabetes displayed improved postprandial glucose control in response to a low-GI diet, but they are not completely concordant with the OmniCarb trial, a similar intervention involving manipulation of GI within a dietary intervention in which plasma insulin concentrations were not influenced by GI [11]. While low-GI diets have clinical utility in individuals with type 2 diabetes [27], our findings support the hypothesis that replacing high-GI foods for low-GI foods might also be useful for improving indices of glycemic control in people with traits of metabolic syndrome, thereby potentially reducing the risk of progression to type 2 diabetes and/or cardiovascular disease [28].

The results from the 24-h continuous glucose monitoring add important context to our postprandial findings. We report that while the mean 24-h glucose concentrations decreased comparatively in both groups following MED-HEPs, changes in the indices of glycemic variability favored the low-GI group. While there is currently a limited consensus on the relative importance of different indices of glycemic variability [29], the consistent improvement observed in the low-GI group across classical and contemporary indices strongly suggests a more stable blood glucose profile after the low-GI diet. The classical indices include the mean amplitude of glucose excursions, mean absolute glucose, and standard deviation, while the contemporary indices include lability index and continuous overall net glycemic action. Mechanistic support for the relevance of glycemic variability can be found in research demonstrating that acute glucose excursions induce oxidative stress to a greater degree than sustained hyperglycemia [6]. However, the clinical relevance of reducing blood glucose excursions remains unresolved, with some [30], but not all [31] evidence supporting glycemic variability as an independent risk factor for overall mortality and cardiovascular diseases. Assessment of glucose variability in individuals at risk for type 2 diabetes, such as our participants, may be particularly important as perturbations in glucose homeostasis may be an early manifestation of glucose dysregulation [32].

We chose to assess the effects of GI in the context of a MED-HEP because the MED-HEP has been linked with a reduced progression to type 2 diabetes in at-risk populations [33]. Indeed, the MED-HEP appears to be particularly effective in improving glycemic control relative to other dietary patterns such as DASH which may be comparatively more effective in reducing blood pressure [34]. Components of the MED-HEP, such as abundant monounsaturated fatty acids in olive oil, may also particularly improve postprandial glycemia via an improved postprandial insulin sensitivity [35].

Strengths of the MEDGI-Carb study include strong clinical design features such as randomization, blinding of the analytical procedures, and an appropriate sample size with respect to effect size and power based on previous research. Further, the robustness of the primary manipulated variable (GI of the carbohydrate foods) was ensured through independent analyses to ensure that the GI of the carbohydrate foods were sufficiently different between the low- and high-GI groups, which had been intended but was difficult to achieve in previous studies. A potential limitation stems from our study sample consisting almost entirely of participants of Caucasian ethnicity. Certain populations, such as Asian individuals, display unique metabolic characteristics that may predispose them to benefit more from the interventions posited to influence the postprandial carbohydrate metabolism [36]. Therefore, care should be taken that these findings are not overgeneralized to people from other racial groups. In addition, our study was tailored to assess the metabolic effects of the dietary intervention but it was not powered for a duration and sample size that would allow for the evaluation of the impact of the diets on clinical events. Lastly, we cannot discount that the health-promoting effects of GI reduction may be greater in the context of a higher-carbohydrate pattern, such as a typical ‘dietary approaches to stop hypertension’ HEP [37], compared with a lower-carbohydrate diet, such as a MED-HEP [38]. Therefore, future studies should investigate both the potential modulation of postprandial glycemia by the monounsaturated fats commonly found in MED-HEPs and assess the potential GI-induced differences from MED-HEP varieties featuring a higher percentage of daily energy from carbohydrates.

## 5. Conclusions

In conclusion, the acute superiority in the indices of postprandial glucose control of participants who emphasized low-GI foods relative to those who emphasized high-GI foods is sustained and was further amplified over time in the context of a background MED-HEP. However, this amplified difference over time is primarily due to a worsening of postprandial glucose control among participants whose diets emphasized high-GI foods. Unexpectedly, the higher insulin responses to consuming high-GI versus low-GI meals at baseline were not apparent after adopting the MED-HEP. Consuming a MED-HEP was sufficient to reduce daily glycemia, but only the low-GI diet resulted in improvements in the indices of daily glycemic variability. Collectively, these findings demonstrate the relevancy of GI within a mixed diet with healthy features resembling those of a traditional Mediterranean-style diet among non-diabetic individuals. Since low-GI foods are an inherent element of a traditional Mediterranean diet [39], our findings suggest that low-GI foods may contribute to the health benefits seen from the MED-HEP.

## Figures and Tables

**Table 1:** Fasting clinical characteristics of participants at screening.

Demographic Characteristics	Low-GI (*n* = 102)	High-GI (*n* = 111)
Age at randomization (years)	55 ± 10	55 ± 11
Female *n* (%)	55 (53.9%)	64 (57.7%)
Weight (kg)	92 ± 14	88 ± 14
BMI (kg/m^2^)	31.1 ± 3.1	30.3 ± 3.0
Waist Circumference (cm)	106 ± 8	105 ± 9
**Metabolic characteristics**		
Glucose (mmol/L)	5.4 ± 0.6	5.4 ± 0.7
Total cholesterol (mmol/L)	4.9 ± 1.0	5.0 ± 0.9
Triglycerides (mmol/L)	1.3 ± 0.6	1.3 ± 0.6
HDL (mmol/L)	1.3 ± 0.4	1.3 ± 0.4
LDL (mmol/L)	3.2 ± 0.8	3.3 ± 0.7
Systolic blood pressure (mm Hg)	126 ± 14	126 ± 14
Diastolic blood pressure (mm Hg)	82 ± 9	83 ± 8

Data are means ± SD. There were no statistically significant differences at baseline.

## Data Availability

The data presented in this study are available on request from the corresponding author.

## Supplementary Materials

The following supporting information can be downloaded at: https://www.mdpi.com/article/10.3390/nu14030706/s1, Figure S1: Meal- and oral glucose tolerance testing day schematic, Figure S2: Insulin and glucose elevations above fasting concentrations in response to low-GI and high-GI Breakfast and Lunch Meals During 8-h Meal Glucose Tolerance Tests at Baseline and After a 12-week Dietary Intervention, Figure S3: Insulin and Glucose Postprandial Areas Under the Curve in Response to low-GI and high-GI Meals During 8-h Meal Glucose Tolerance Tests at Baseline and After a 12-week Dietary Intervention, Figure S4: 1-h and 2-h glucose and insulin responses at to a 2-h oral glucose tolerance test (75 g glucose bolus), Table S1: Food Composition of Standardized Meal Glucose Tolerance Test Meals, Table S2: Dietary Composition Before And After A low- And high-GI Mediterranean Dietary Intervention, Table S3: Effect of low- versus high-GI Healthy Eating Patterns on Risk Factors for Type 2 Diabetes.
