# Supplementary material for: Differential Glycemic Effects of Low- versus High-Glycemic Index Mediterranean-Style Eating Patterns in Adults at Risk for Type 2 Diabetes: The MEDGI-Carb Randomized Controlled Trial
Source: Nutrients. 2022 Feb 8;14(3):706. doi: 10.3390/nu14030706 (PMC8838655; doi:10.3390/nu14030706)
Supplement: Supplementary file 1 [file nutrients-14-00706-s001.zip › nutrients-1528915-supplementary.pdf]

ONLINE-ONLY SUPPLEMENTAL MATERIAL

**Supplemental Table S1.** Food Composition of Standardized Meal Glucose Tolerance Test Meals			
**Low-GI**		**High-GI**	
**Breakfast**	**Quantity**	**Breakfast**	**Quantity**
Flat bread	75 g	Cornflakes	30 g
Olive Oil, Extra Virgin	10 g	Olive Oil, Extra Virgin	18 g
Eggs, whole, raw	50 g	Eggs, whole, raw	50 g
Ham, dry cured (country style)	38 g	Ham, dry cured (country style)	85 g
Apple, fresh without skin)	150 g	Apple, fresh without skin	150 g
milk, 1% fat or lowfat, lactose free	244 g	milk, 1% fat or lowfat, lactose free	244 g
		Bread, wholegrain	24 g
**Lunch**		**Lunch**	
Spaghetti noodles, white,	90 g	Rice, jasmine	88 g
Chicken, breast, skinless	58 g	Chicken, breast, skinless	70 g
Tomato sauce, plain, regular	50 g	Tomato sauce, plain, regular	50 g
Olive Oil, Extra Virgin	10 g	Olive Oil, Extra Virgin	10 g
Broccoli, cooked from frozen	90 g	Broccoli, cooked from frozen	90 g
Carrots, cooked from frozen	100 g	Carrots, cooked from frozen	100 g
Apple, fresh, without skin	150 g	Apple, fresh, without skin	150 g

Supplemental Table S2. Dietary composition before and after a low- and high-GI Mediterranean dietary intervention

	**Baseline**			**Post-intervention**		
	**Low-GI**	**High-GI**	**p-value**	**Low-GI**	**High-GI**	**p-value**
**Energy (kcal/day)**	1923±508	1939±503	0.845	2344±609*	2257±463*	0.314
**Proteins (% of TE)**	16.6±3.0	16.6±3.1	0.914	18.7±3.9*	18.6±2.7*	0.877
**Fat (% of TE)**	38.4±6.2	38.1±5.7	0.744	36.0±6.0	35.6±5.8*	0.632
**SFA (% of TE)**	13.2±4.0	13.5±3.9	0.608	9.6±2.8*	9.8±3.2*	0.764
**MUFA (% of TE)**	15.3±3.5	14.3±3.0	0.067	16.3±4.1*	16.7±3.3*	0.531
**PUFA (% of TE)**	5.6±2.4	5.7±2.7	0.814	4.8±1.6*	4.8±1.4*	0.994
**Cholesterol (mg/day)**	291±141	292±133	0.983	328±208	341±219	0.684
**Carbohydrates (g/day)**	219.6±64.4	222.5±62.4	0.775	259.0±46.6*	253.1±34.5*	0.373
**(% of TE)**	42.9±6.4	43.2±6.0	0.791	42.5±6.4	43.0±6.6	0.585
**Starch (g/day)**	151.5±48.5	151.0±50.6	0.952	192.7±50.5*	184.9±54.2*	0.350
**Sugars (% of TE)**	13.1±5.3	13.9±5.5	0.363	11.0±6.5*	12.0±7.3*	0.343
**Fiber (g/day)**	19.4±7.5	20.2±5.9	0.489	32.4±7.1*	30.8±5.8*	0.113
**(% of TE)**	2.0±0.6	2.1±0.6	0.289	2.8±0.6*	2.8±0.6*	0.786
**Glycemic index (au)**	59.0±6.3	59.4±5.6	0.299	46.8±3.1*	66.2±4.7*	<0.0001
**Alcohol (g/day)**	9.1±12.8	8.0±11.9	0.564	2.3±4.4*	3.7±7.0*	0.109

*Statistically significant, $p < 0.05$.

Supplemental Table S3. Effect of Low- versus High-GI Healthy Eating Patterns on Risk Factors for Type 2 Diabetes									
Health Parameter	**Low-GI**			**High-GI**				**Pooled**	
	**Baseline**	**Post (Week 12)**	**Δ**	**Baseline**	**Post (Week 12)**	**Δ**	**p-value (Group*Time)**	**Δ**	**p-value (Time)**
**Glucose (mmol/L)**	5.7 ± 0.1	5.6 ± 0.1	0.0 ± 0.1	5.8 ± 0.1	5.7 ± 0.1	−0.1 ± 0.1	0.48	0.0 ± 0.0	0.45
**Insulin (pmol/L)**	100.2 ± 6.9	98.3 ± 7.1	−1.9 ± 6.9	103.7 ± 6.8	100.0 ± 7.2	−3.6 ± 7.2	0.84	−2.8 ± 5.6	0.62
**HbA1c (%)**	5.43 ± 0.04	5.40 ± 0.04	−0.02 ± 0.02	5.47 ± 0.04	5.42 ± 0.04	**−0.051 ± 0.025***	0.33	−0.04 ± 0.02	0.07
**HOMA:IR**	3.71 ± 0.28	3.63 ± 0.29	−0.08 ± 0.29	3.97 ± 0.28	3.74 ± 0.30	−0.22 ± 0.30	0.70	−0.15 ± 0.24	0.52
Data are mean ± SEM. *Statistically significant, p < 0.05.									

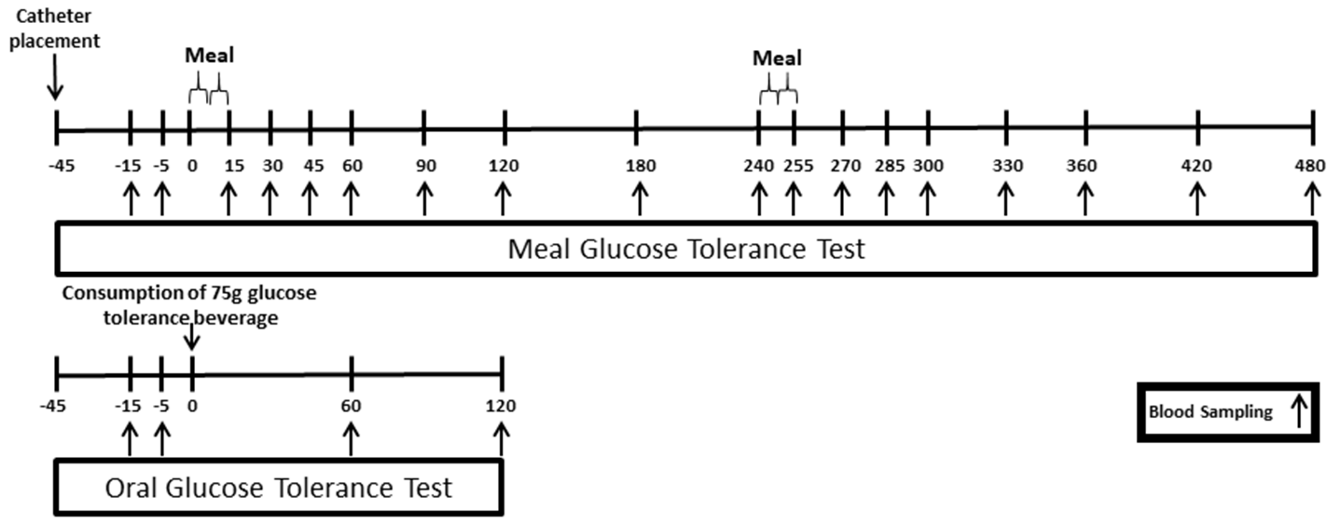

Supplemental Figure S1 - Meal- and oral glucose tolerance testing day schematic.

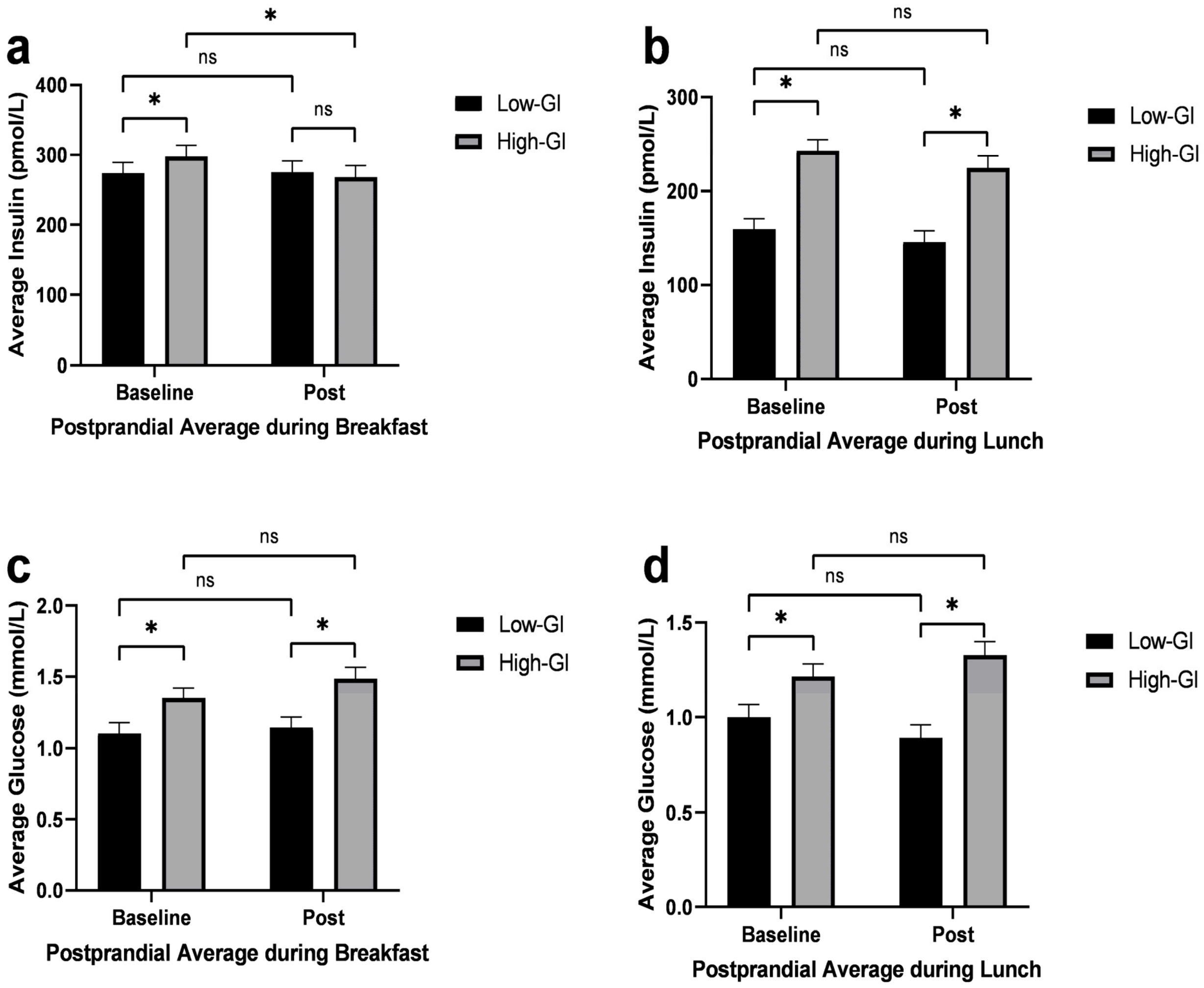

Supplemental Figure S2 - Insulin (a, b) and glucose (c, d) elevations above fasting concentrations in response to Low-GI and High-GI breakfast (a, c; served at timepoint=0min) and lunch (b, d; served at timepoint=240min) meals during 8-h meal glucose tolerance tests at baseline and after a 12-week dietary intervention. Data are LSmeans ± SEM. *Statistically significant, $p < 0.05$.

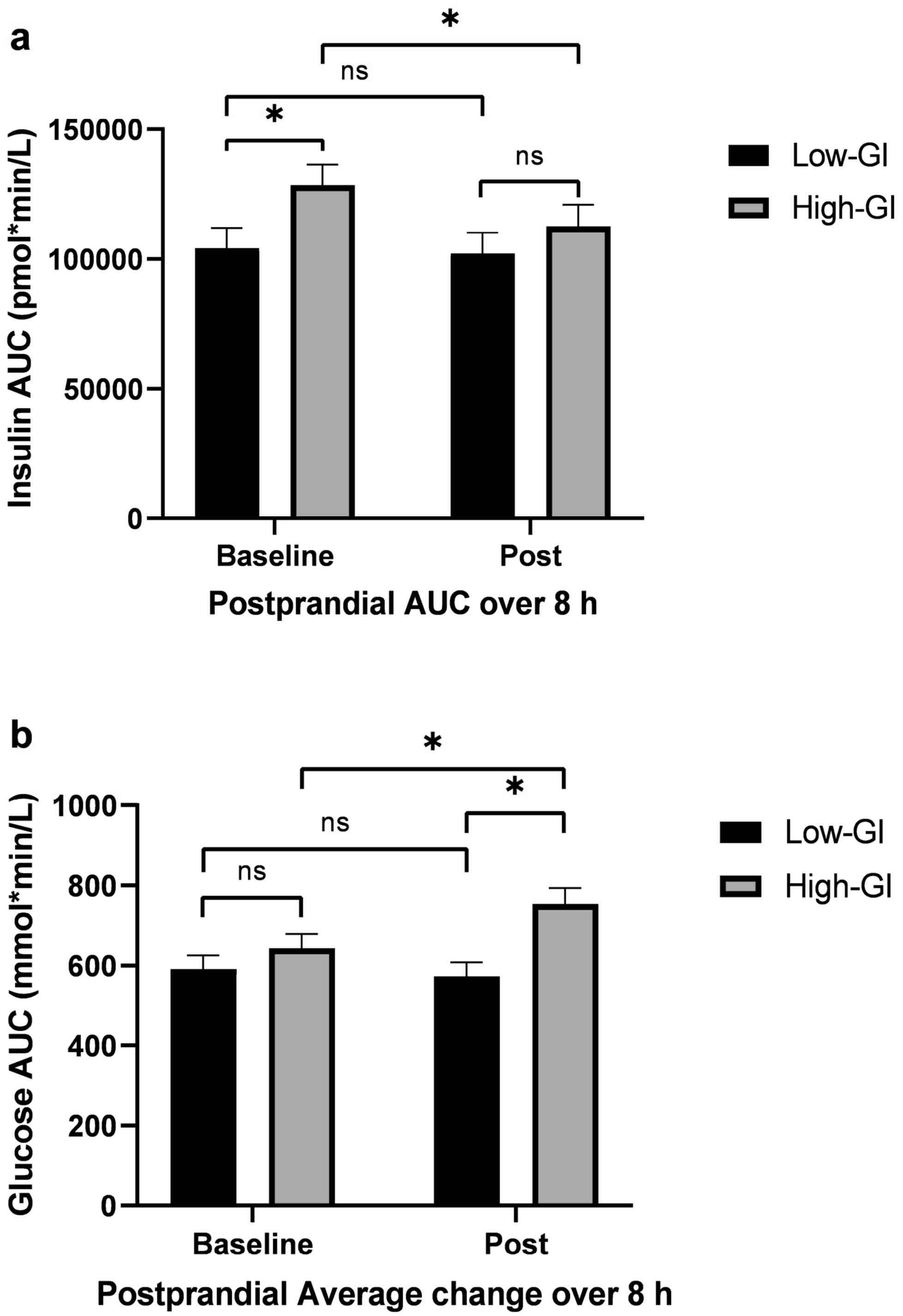

Supplemental Figure S3 – Insulin (a) and glucose (b) postprandial areas under the curve (AUC) in response to Low-GI and High-GI meals during 8-h meal glucose tolerance tests at baseline and after a 12-week dietary intervention. Data are LSmeans ± SEM. *Statistically significant, $p < 0.05$.

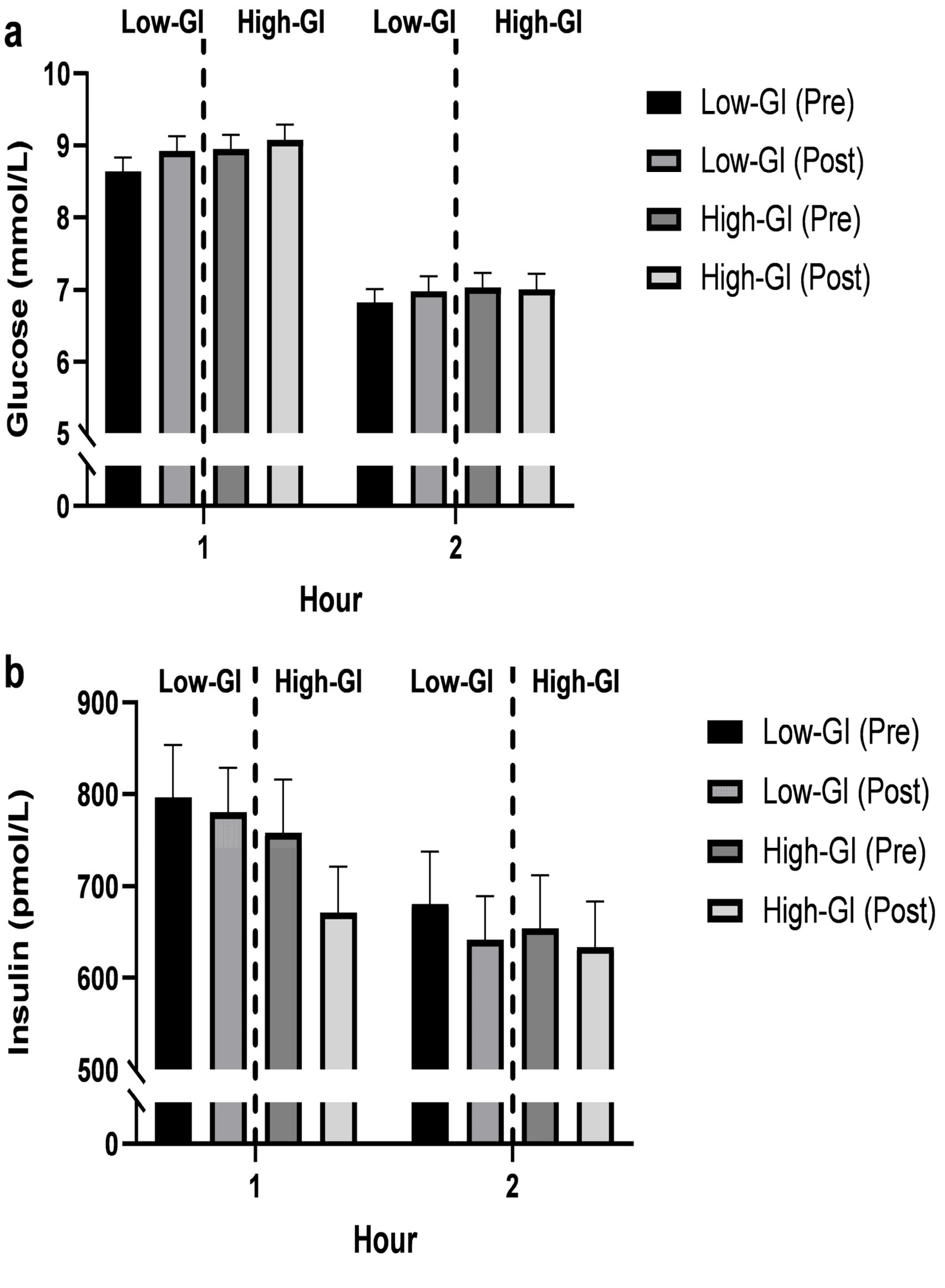

Supplemental Figure S4 – 1-h and 2-h glucose and insulin responses at to a 2-h oral glucose tolerance test (75g glucose bolus). Data are LSmeans ± SEM. *Statistically significant, $p < 0.05$.
